# Has the establishment of national parks improved nature-based tourism experiences? Evidence from social media data

**DOI:** 10.1371/journal.pone.0343256

**Published:** 2026-03-20

**Authors:** Yi Li, Yitong Yang

**Affiliations:** 1 College of Economics and Management, South China Agricultural University, Guangzhou, Guangdong, China; 2 Guangdong Provincial Key Laboratory of Philosophy and Social Sciences, Rural Revitalization Laboratory, Guangzhou, Guangdong, China; University of Ferrara, ITALY

## Abstract

National parks are vital institutions for conserving biodiversity and preserving biocultural heritage that safeguard exceptional natural resources and sustain key cultural ecosystem services (CESs), such as aesthetic appreciation, nature-based recreation, and environmental education. However, the essentially subjective and intangible characteristics of CESs are obstacles to effectively collecting data and measuring their diverse values. This study developed a supply–support–demand framework for analyzing nature-based tourism in national parks, using Giant Panda National Park, one of China’s earliest national parks, as the case study site for the years 2009–2023. To investigate the impact of national park establishment, we employed a difference-in-differences (DID) approach to assess its effects on counties within national parks. We found that tactile and visual impressions play a key role in shaping visitors’ experiences, whereas olfactory, gustatory, and auditory perceptions exhibit cooccurring, supportive influences on visual and tactile impressions, as revealed through the rich expressions captured in social interactions. The establishment of national parks has the potential to enhance nature-based tourism experiences by increasing local fiscal expenditure and expanding the tourism industry. The effects exhibit clear regional heterogeneity and differences in policy intensity. At the regional level, Sichuan and Shaanxi, regions with relatively stronger infrastructure and resource endowments, show better improvement in nature-based tourism experiences, whereas Gansu shows an insignificant effect. In terms of policy intensity, stronger interventions are accompanied by stricter ecological protection constraints. As a result, the improvement in nature-based tourism experiences is significantly greater in the low-intensity policy group than in the high-intensity policy group.

## Introduction

Ecosystems are essential to human well-being, providing not only material and life support but also leisure, recreation, and aesthetic enjoyment [1]. CESs are essential for maintaining ecosystems and traditional communities [2]. Additionally, they are an effective means of disseminating ecological conservation ideals, which are directly related to human welfare [3]. Nature-based tourism is considered a primary means of accessing CESs and plays a vital role in generating economic income to support regional economic development; it also addresses the growing demand for green consumption at the community, regional, and national levels [4]. Nature-based tourism systems centered on national parks (NPs) are vital hubs for the provision of CESs and are typical examples of natural destinations. However, CESs are characterized by subjectivity, intangibility, and incommensurability, which hinder the full recognition, understanding, and assessment of their multiple values [5,6]. Geographic remoteness, unclear administrative boundaries, and insufficient smart monitoring systems [7,8] contribute to spatial and temporal barriers in destination management, including limited accessibility, fragmented data ecosystems, and unmonitored visitor flows [8,9]. Additionally, comprehensive information on tourism experiences, particularly with respect to large spatial scales and the depth of experiential perception, is lacking [10]. It is challenging to integrate and utilize such information effectively in ecological management and industrial development because of the lack of extensive information on tourism experiences [10].

With the rapid growth of the internet, new ways of interaction and experiences sharing between tourists and destinations have been enabled by social media, leading to a new tourism ecosystem [11]. By sharing their travel experiences and feelings, contemporary tourists actively construct experiential narratives on social media [12,13]. Such experience sharing produces rich user-generated content (UGC) that provides insights into the cultural values of nature and supports the sustainable management and conservation of natural heritage [14,15]. With the rise of UGC, new ways of searching for travel information have emerged, influencing the motivation, behavior, and consumption patterns of tourists [16]. Nevertheless, the prevailing research methodologies remain constrained by traditional survey instruments (e.g., questionnaires and interviews), which, despite their prevalence, have inherent limitations, including temporal inefficiencies, response biases, and spatial constraints [6].

This study employed computational text mining techniques to analyze social media-derived UGC, focusing on (1) interpreting CESs through sensory experience data generated from human–nature interactions and (2) investigating the mechanism through which NP establishment enhances nature-based tourism experiences, particularly multilevel impacts on destination selection preferences and geographical impression formation [1]. This study aimed to examine the value of NPs and to provide insights for enhancing nature-based tourism and promoting sustainable NP management. This study makes three original contributions to the field. First, this study presents a framework for assessing policy impact by systematically examining the multilevel effects of NP establishment on nature-based tourism experiences in counties within national parks (WNP counties). By examining how protected area governance improves tourism experiences, this study provides a theoretical basis for evaluating conservation policy effectiveness. Second, this study reveals tourists’ emotional and perceptual experiences by visualizing social media text data. This study deepens the understanding of interactions between tourists and destinations by interpreting CESs from multiple dimensions. From a methodological perspective, this study offers a framework that can inform efforts to enhance nature-based tourism experiences and provides practical insights for destination management and policy-making. Third, this study quantitatively examines how the establishment of NPs improves nature-based tourism experiences and reveals regional variations in its effects. These findings offer real evidence for flexible management strategies and provide specific recommendations to help create sustainable development policies for protected areas.

## Literature review

### Research progress on nature-based tourism experience valuation

The conceptualization of nature has evolved from an anthropocentric dichotomy (“human-nature opposition”) to a relational ontology, reflecting epistemological advancements in the ecological sciences and environmental philosophy [5]. Contemporary scholarship redefines natural systems as dynamic assemblages that incorporate cultural value, intangible value, and spiritual value, moving beyond the concept of “pristine wilderness” [5]. Through stewardship practices, humans ascribe profound cultural value to biophysical environments, transforming them into sociocultural artifacts that embody historical consciousness and spiritual heritage [17]. These positive interactions with nature promote both inner peace and self-awareness and spark positive emotions such as excitement and joy. These benefits contribute significantly to the enhancement of overall well-being and health [18–20]. Individuals are increasingly seeking nature-based tourism destinations to relieve cognitive fatigue and restore well-being through purposeful immersion in natural environments [21]. Nature-based tourism activates two mental processes: (1) it refreshes the mind and creates positive feelings from calming experiences [22], and (2) it improves the connection between people and nature, which increases how much people value a place, leading to stronger loyalty to the destination and affecting overall satisfaction. Furthermore, factors such as the quality of the tourism experience, the extent of emotional resonance, and perceived price fairness significantly shape visitors’ satisfaction with nature-based tourism, which in turn promotes environmentally responsible behavior [21].

While supply-side actors increasingly prioritize nature-based tourism, the development and management of cultural and spiritual values remain insufficient, revealing an urgent need for research to enhance destination attractiveness through value-based strategies [5]. NPs are biocultural repositories, preserving valuable ecological assemblages and safeguarding both tangible and intangible heritage, making them prime destinations for nature-based tourism. Grounded in an ecosystem value framework, the construction of NPs systematically safeguards the following tripartite value dimensions inherent to natural ecosystems: instrumental value [23,24], intrinsic value [23–25], and relational value [26,27]. Within this framework, nature-based tourism has become a key pathway for the sustainable development of NPs by providing financial support for ecological conservation [28] and fostering tourism–territory linkages that translate the relational value of ecosystems into socioeconomic benefits and support the development of distinctive local tourism services. Regional examples include the ecostewardship model of the Shennongjia NP, which follows a value chain of “conservation-tourism-community revitalization” [29], and the Pudacuo NP, which has demonstrated exceptional efficacy in poverty alleviation through nature-based tourism initiatives [30]. The documented precedents validate NPs’ dual responsibility for conservation and development through their biospheric management and geoeconomic facilitation. However, although studies have focused on ecological [29,31] and social dividends [30–33], significant gaps remain in terms of clarifying methods to enhance tourist experiences through facility development, landscape management, and ecocultural integration. More extensive empirical and theoretical analysis are still needed to clarify how enhanced tourist experiences can support local development and sustain the long-term viability of National Parks. Tourists’ experiences correlate with public attention to NPs [34] and directly predict environmentally responsible behavior [35]. Consequently, elucidating experience optimization frameworks that amplify the value-actualization mechanisms of CESs through nature-based tourism has emerged as a critical scientific imperative. This inquiry provides a more scientifically grounded basis for the future establishment of similar NPs.

### Social media analytics in nature-based tourism experience valuation

As an emerging post hoc research tool, social media data are being increasingly used to assess tourist experience dimensions and preference structures, and their methodological advantages, including ease of data acquisition and large sample sizes, make them valuable complements to conventional questionnaires and interviews [8,36]. While field-based approaches (questionnaires/interviews) remain foundational for tourism experience analysis [37–39], they require significant temporal and resource investments. For example, comparative analyses of social media data from Instagram, Twitter, and Flickr against official visitor statistics validate their efficacy in demonstrating visitation intensity and destination appeal in the establishment of NPs [40]. The geotagged metadata of Flickr photos, which allows for high-accuracy inferences of visitors’ residences, have been effectively used to analyze spatiotemporal patterns of recreation across different demographic groups [41].

The academic community has increasingly adopted social media data analytics to investigate tourist behavior and experiences through geospatial footprints, image sharing patterns, and digital engagement metrics. Platforms such as Flickr and Instagram provide georeferenced imagery that enables spatial behavior analysis [42,43], such as mapping the distribution of NP visitors for optimized tourism management [44]. Wikipedia traffic metrics serve as predictive indicators of destination interest, offering proactive signals for tourism governance, whereas Instagram content reflects postvisitation engagement [45]. Complementary Google Trends analyses reveal significant correlations between search keyword frequency and public interest in protected ecosystems [15,46]. This methodological paradigm has become a mainstream analytical approach that uses social media APIs, crowdsourced data, and computer vision tools such as Google Cloud Vision to automatically analyze photo content and extract emotional and experiential information, thereby demonstrating the potential of social media image analytics for assessing visitor interests and affective responses in conservation settings [47–49]. While visual social media analytics (for example, geotagged photography) have increased experience evaluation capabilities, textual metadata provide greater granularity in decoding tourists’ cognitive-emotional processing [6]. This study employed textual analytics to decode travelers’ unique cognition and preference patterns while simultaneously revealing their diverse emotional experiences. These insights contribute to the development of more informed decision-support frameworks for place stewardship and tourist engagement.

### Value cocreation mechanisms in tourist experiences: a social media analytics framework

Contemporary scholarship theorizes the tourist experience as a multidimensional sociopsychological construct [50] that emerges from the confluence of interdependent factors that collectively shape destination perceptions and evaluative attitudes, reflecting individuals’ affective states throughout service interactions [51]. Scholars have investigated key determinants of enhancing tourism experiences, including individual differences in cultural background [52] and nationality [53], travel expectations [54], emotional states [55], and participatory engagement and cultural interactions [56]. The research paradigm is anchored predominantly in individual-level analyses of behavioral patterns, demographic attributes, and instrumental facilitators, with limited examination of dyadic interactions between visitors and tourism destinations. Moreover, insufficient scholarly attention has been given to cultural values and the management of spiritual dimensions in nature-based tourism destinations [5].

The postdigital transformation era has resulted in social media evolving into interaction spaces, creating novel value cocreation pathways that align with virtualized experience economies [57]. The increasing popularity of social media has attracted the attention of academics and business professionals alike [58]. More users share experiential content on these platforms, and platform features actively increase these shared experiences [59]. Social media now significantly affect the tourism sector [15,60], guiding tourists’ destination choices and behavioral decisions. As the primary platform for tourist interactions and information exchange, social media fundamentally enable value cocreation processes in contemporary tourism [61–63].

As an infrastructural pillar of value cocreation ecosystems, social media enable synergistic resource integration and information dissemination [64]. Research predominantly applies service-dominant (S-D) logic frameworks, conceptualizing users as active coproducers who shape peer consumption decisions through participatory engagement—a strategic imperative for enterprise marketing [65–68]. However, S-D logic applications systematically underrepresent customer centrality in cocreative processes [65]. In response, research has adopted customer-dominant logic paradigms to decode tourists’ value cocreation behaviors, yielding actionable insights for destination marketing optimization [67,68]. Advanced methodologies, including partial least squares structural equation modeling, have identified critical determinants of travel experience sharing, informing strategies to mitigate behavioral barriers in tourist engagement [69]. Social media data serve as a perceptual proxy for tourist experiences, providing actionable insights for destination governance [70]. Dolan et al. analyzed 1,038 social media posts and user interaction datasets to identify platform-dependent participation mechanisms, revealing contextual moderating effects on UGC and engagement behaviors on Facebook and Instagram [71]. Nevertheless, the inherent experiential essence of tourism [72] demands a stronger scholarly focus on experiential value cocreation mechanisms [73–75]. Current research inadequately addresses the capacity of social networks to mediate tourist destination interactions for experiential value realization, thus highlighting a theoretical and practical gap.

## Theoretical framework

This study advanced an integrative supply–support–demand framework to decode how the construction of China’s NPs shapes nature-based tourism experiences. Building upon Gunn’s foundational tourism system model [76] and Wu Bihu’s tripartite extension (demand–supply–support) [77], we adapted and refined the framework to align with the contextual dynamics of China’s NP establishment. Specifically, this study clarifies and extends the functional linkages among the three subsystems by making explicit how the mechanisms operate under the institutional context of China’s NP establishment. In adapting the original supply–support–demand structure, we introduced a digital-era enhancement to the demand subsystem, in which tourists, potential tourists, and online platforms jointly produce extensive shared UGC through interactive processes. By incorporating a platform-mediated and participatory layer of demand formation, these social media-enabled value cocreation pathways alter the conventional model and change the way experiential value is produced and disseminated within the tourism system. The protected area tourism system, the National Park County Nature Tourism Supply Nexus, synthesizes unique ecocultural capital and nature-based amenities, which act as important reservoirs for the strategic provision of vital natural and cultural assets. The National Park Policy Empowerment Nexus, which includes rules and local management, helps support nature tourism by considering local social, environmental, and community factors. Using social media channels to coproduce multimodal UGC, including service interactions, sensory inputs, and emotive engagements, the “National Park County Nature Tourism Demand” Nexus, the demand subsystem, orchestrates tourist-to-participant conversion through digital intermediation. This platform-mediated coproduction mechanism increases the dynamic propagation and participatory valorization of the ecocultural assets of protected areas, enabling tripartite stakeholder cocreation (destination stewards, potential visitors, and engaged visitors) within the nature-based tourism value chain.

**Fig 1:**
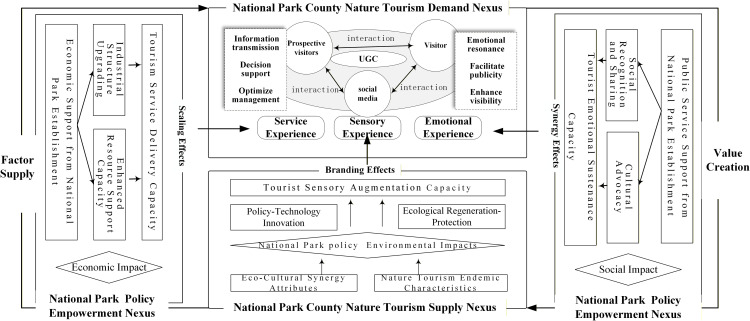
Theoretical Framework.

Within this conceptual framework (Fig 1), this study identifies three main mechanisms through which national park establishment alters local nature-based tourism systems: (1) branding effects, (2) synergy effects, and (3) scaling effects.

### Branding effects

Operating as intangible institutional capital, the NP’s brand captures latent market value by synthesizing environmental and governance resources. Methodical analysis of this branding strategy indicates how well it can inspire value-added development in auxiliary industries. At its core lies the NP’s ecological genotype—a distinctive configuration of ecosystem integrity and institutional efficacy actualized through policy innovation, technological advancement, and conservation interventions. These combined efforts improve important environmental quality measures, such as air cleanliness, the beauty of the landscape, and the variety of plant and animal life, all of which make visitors’ nature-based tourism experiences more enjoyable. Increased ecological genotypes increase destination competitiveness in two ways: first, by providing biophysical foundations for sensory satisfaction through improved air quality, visual landscapes, and species richness; second, by establishing destination brand equity through ecological differentiation. The resultant visitor loyalty and organic social media dissemination of positive experiences accelerate reputational diffusion, crystallizing destinations’ ecoaesthetic brand identity. In the long term, this environment-based branding effect helps attract more visitors and investment, thereby promoting the sustainable economic development of national parks and their surrounding nature-based tourism regions. The following hypothesis is thus proposed:

H1: The establishment of NPs positively improves nature-based tourism experiences in WNP counties relative to those in counties outside NPs (ONP counties).

### Synergy effects

The establishment of NPs enhances emotional experiences in nature-based tourism within WNP counties through social impacts. Tourists typically hold high expectations regarding the infrastructure and environmental conditions of the destination [78]. Article 44 of the National Park Law of China establishes a funding guarantee system for public finance, providing a fundamental institutional basis for strengthening the provision of public services within NPs. Against this backdrop, NP establishment relies on institutionalized fiscal spending to effectively improve the quality and accessibility of public services, including infrastructure and environmental education, which play a key role in meeting tourists‘ expectations and enhancing their experiences. Effective public service provision not only lays a solid foundation for positive emotional experiences among visitors, but also, with the support of fiscal expenditure, enables the systematic development and dissemination of local culture. This process gives rise to emotionally resonant narratives and interactive experiences, offering tourists deeper forms of recreation that combine authenticity with warmth. These emotional experiences are key factors in creating value in tourism, leading to lasting memories [72] that encourage people to engage online through social media, which builds loyalty unrelated to purchases. Thus, the establishment of NPs supports emotional experiences in nature-based tourism within WNP counties by strengthening fiscal expenditure to secure the material and cultural conditions required for experience enhancement. The following hypothesis is therefore proposed:

H2: Increases in local fiscal expenditure, proxied by general budget expenditure, constitute one potential pathway through which NP establishment enhances nature-based tourism experiences.

### Scaling effects

NP establishment has scale effects on service systems for nature-based tourism in WNP counties by promoting resource concentration. Beyond supporting local development, it strengthens the capacity of tourism service facilities, including accommodation, catering, and transportation, by driving the upgrading of related industries such as tourism and retail. Improving tourism service capacity implies a more diverse, reliable, and accessible supply of essential services. Within tourism experience theory, these services constitute the supportive dimension of the tourism experience [79], serving as a fundamental determinant of overall visitor satisfaction, representing core visitor benefits, and ultimately forming the basis of information transmission on social media [80]. Accordingly, the tourism industry scale expansion induced by NP establishment improves tourists‘ basic consumption experiences at destinations by systematically upgrading the quality of this supportive experiential dimension. Therefore, this paper formulates the third theoretical hypothesis to be tested:

H3: NP establishment can enhance tourism experiences by promoting the expansion of the local tourism industry.

## Data, methods, and models

### Data

**Fig 2:**
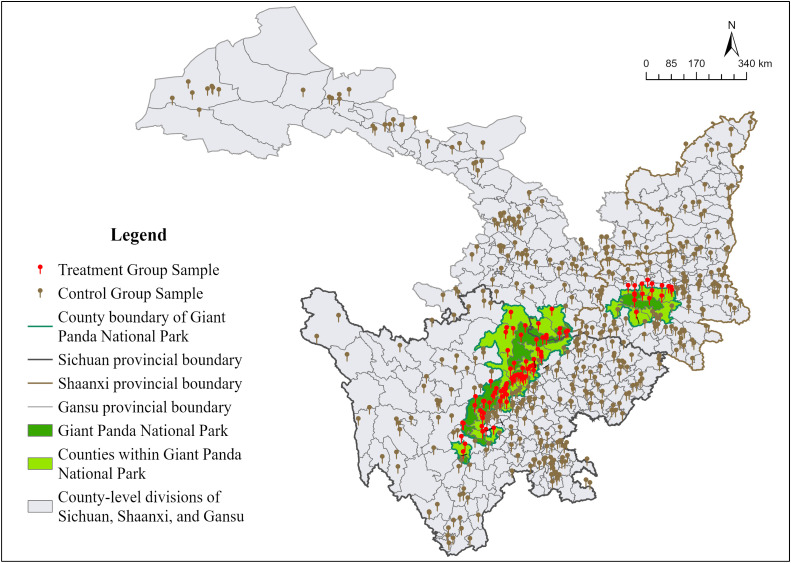
Distribution of the Treatment and Control Groups.

Giant Panda National Park (GPNP), the world’s only sanctuary for giant panda habitats, possesses not only abundant ecological resources and rare species but also exceptional global recognition. As one of the Chinese inaugural NPs, it serves as a paradigmatic model for reconciling ecological conservation with tourism development. By analyzing its effects on tourists’ impressions, we can learn how unique ecological resources shape visitors’ affective responses, which can guide the development of better preservation strategies for similar NPs. The Giant Panda National Park (GPNP) was officially approved and launched as a national park construction pilot in 2017. The area of GPNP spans 27,134 square kilometers across 12 prefectures and 30 counties in Sichuan, Shaanxi, and Gansu Provinces. For this study, based on formally designated Grade A natural scenic sites (2009–2023) across Sichuan, Shaanxi, and Gansu Provinces (N = 554), we defined the treatment group as sites within county-level administrative units overlapping the GPNP boundaries (as delineated by the cyan county borders in Fig 2). Conversely, the control group comprises all remaining Grade A sites in the three provinces situated outside these national-park-designated county boundaries (visualized as brown symbols distributed across nonpark areas in Fig 2). As shown in Fig 2 (generated via GIS), the geographical distribution clearly differs between the treatment and control groups: 100 sites located in WNP counties constitute the treatment group, whereas 454 sites in ONP counties form the control group. While samples from the control group are dispersed in nonpark locations across the three provinces, samples from the treatment group are shown in Fig 2 and grouped inside WNP county limits.

The research data are derived from three primary sources. First, a web crawler was used to collect publicly shared social media content from Dianping.com, the largest crowdsourced online review platform in China. The captured UGC includes user IDs, timestamps, and textual narratives. As the earliest domestic platform dedicated to local lifestyle information and transactional services, Dianping.com [81] is among the world’s pioneering third-party consumer evaluation websites. By 2015, it had more than 200 million monthly active users scattered throughout approximately 2,500 cities worldwide, with more than 100 million user-generated evaluations. Second, data were extracted using Octoparse from Tianyancha, a comprehensive corporate information system. This platform provides comprehensive business intelligence, particularly operational data from hotels. Third, county-level socioeconomic indicators were sourced directly from national and provincial statistical yearbooks and the China County Statistical Yearbook.

### Methods

In this study, natural language processing (NLP) techniques were employed using PyCharm to extract visitors’ nature-based tourism experiences from UGC and to visualize their semantic structure. The methodological workflow consisted of three stages: textual analysis, sentiment analysis, and experience-dimension extraction with co-occurrence network construction. (1) Textual analysis. The Jieba library was used to tokenize Chinese sentences into units that could be analyzed before segmenting all review texts. A customized stopword list was then compiled by merging multiple stopword dictionaries to remove meaningless functional words and improve the accuracy of subsequent analyses. (2) Sentiment analysis. Social media reviews were then analyzed with respect to sentiment via the SnowNLP package, a Python-based NLP toolset trained on extensive Chinese datasets. The sentiment classification framework operationalizes textual analysis into three discrete affective categories: positive sentiment, neutral sentiment, and negative sentiment. (3) Cooccurrence network. YAKE was used to find more noteworthy terms and perform unsupervised keyword extraction. Afterward, a feature matrix was created, and the word co-occurrence matrix was calculated using a TF–IDF vectorizer to create a keyword co-occurrence network.

To further identify and clarify the mechanisms and causal effects through which NP establishment enhances visitors‘ experiences, this study employs econometric methods for empirical analysis. Specifically, we use a difference-in-differences (DID) approach to examine the impact of national park establishment on nature-based tourism experiences. All empirical analyses are conducted using Stata 17.

### Models

#### Model construction.

This study treats the establishment of NPs as a quasinatural experiment. A DID model was used to assess the causal effects of the policy through the establishment of the GPNP [82]. The regression model was defined via the following established identification procedures:


$$Y_{it}=\beta_{0}+\beta_{1}({\text{Treated}}_{i}\cdot {\text{Post}}_{it})+\lambda \sum {\text{Controls}}_{ct}+v_{i}+\tau_{t}+e_{it}\;$$ (1)

where $Y_{it}\;$ is the dependent variable denoting the natural logarithm of the total number of UGC instances or the count of positive comments for scenic spot *i* in year *t*, which acts as a proxy for the effect of the NP institution pilot on the nature tourism experience; the interaction term ${\text{Treated}}_{i}*{\text{Post}}_{it}\;$ is the product of a treatment dummy and a time dummy, where ${\text{Treated}}_{i}=1\;$ if scenic spot *i* is located in WNP counties and 0 otherwise, and ${\text{Post}}_{it}=1\;$ for years after the 2017 pilot implementation but 0 for years before 2017; ${\text{Controls}}_{ct}\;$ represents a set of time-varying county-level control variables; $v_{i}\;$ and $\tau_{t}\;$ control for scenic-spot fixed effects and time fixed effects, respectively; $\beta_{1}\;$, which is the primary estimable parameter, reflects the net effect of NP establishment on nature-based tourism experiences; and $e_{it}\;$ is the error term.

To further examine the mechanisms through which the establishment of NPs affects nature-based tourism experiences, this study specifies the following regression models, drawing on approaches adopted in previous studies [83,84].


$$M_{ct}=\beta_{0}+\beta_{1}({\text{Treated}}_{i}\cdot {\text{Post}}_{it})+\lambda \sum {\text{Controls}}_{ct}+v_{i}+\tau_{t}+e_{it}\;$$ (2)

In Equations (2), $M_{it}\;$ denotes the mechanism variables, specifically the local fiscal expenditure and the tourism industry scale of county c in year t. All the other variables maintain consistent definitions with Equation (1).

#### Variable definition and description.

Dependent Variable: UGC from Dianping.com was used to evaluate how the establishment of NPs enhances nature-based tourism experiences. For each scenic location and year (2009–2023), two primary indicators were obtained: the total number of reviews (RC), which reflects the location popularity and visitor interaction intensity and serves as an indicator of tourist engagement, and the number of positive sentiment reviews ($\;SA_{\text{pos}}\;$), as identified through sentiment classification, which captures visitor satisfaction and the likelihood of recommendation [85]. To minimize scale differences and preserve distributional properties, both variables were log-transformed (lnRC , $\ln SA_{\text{pos}}\;$). To verify the classification accuracy of the SnowNLP sentiment analysis model, we manually annotated a random sample of 3,000 reviews. The model achieved an accuracy of 0.812, which meets the threshold for analytical use [86].

Key Explanatory Variable: To examine how NP establishment enhances nature tourism experiences, the core explanatory variable captures the policy effect of NP establishment, denoted as ${\text{Treated}}_{i}*{\text{Post}}_{it}\;$, which is operationalized as the interaction term between the treatment group and the postimplementation period dummies.

Control Variables: To reduce the potential confounding effects, eight main regional factors affecting nature-based tourism experiences were considered. First, the industrial structure (stru) was an important factor impacting the tourism experience. It is reflected by the percentage of GDP from the service sector, which indicates the level of development of local tertiary industries. Second, GDP per capita reflects the broader economic vitality of a region, which shapes market conditions and service availability and may affect tourism experiences; thus, pcGDP must be included as a control variable. Third, urban per capita disposable income (UrbPCDI) reflects both living conditions and regional purchasing power. Higher income levels correspond to improved service quality and infrastructure, as well as increased travel expenditures. Fourth, total social fixed asset investment (TSFAI) measures capital allocation intensity and economic dynamism. More investment immediately helps to build infrastructure and tourism facilities, influencing the quality of the travel experience. Fifth, resident population size (RPop) is included to account for carrying capacity implications, as demographic endowment influences the distribution of tourism resources. Sixth, the number of full-time teachers in regular secondary schools (RSST) represents the regional educational resource distribution and hence influences the quality of the human capital available for tourism operations. Seventh, secondary industry output (SecInd) captures the level of regional industrialization and potential externalities that may influence the tourist site surroundings. Finally, tertiary sector employment (TertIE) is controlled to explain human capital allocation to service sectors, which influences the quality of nature tourist services provided.

Mechanism Variables: This study employed two mechanism variables to investigate the possible avenues through which NP establishment can improve nature-based tourism experiences. Fiscal expenditure reflects the level of local government spending on public infrastructure and environmental management. The tourism industry scale is proxied by the annual size of the local accommodation sector, which serves as an indicator of the development level of services related to tourism. These mechanism variables help assess whether the construction of NPs potentially contributes to improved visitors’ experiences through channels such as higher fiscal expenditure and a larger tourism industry scale.

#### Descriptive statistics of the variables.

Table 1 provides descriptive metrics for the core variables. The $\ln SA_{\text{pos}}\;$ has a mean of 1.143 (SD = 1.628) and ranges from 0 to 8.569. Comparatively, lnRC shows similar dispersion, with a mean of 1.265 (SD = 1.728) over the observed scale. These findings indicate large variations in the attractiveness and reputation of various scenic areas, revealing major discrepancies in tourist experiences across these locations.

**Table 1:** Descriptive Statistics of the Key Variables.

Variable Name	N	Mean	SD	Min	Max
$\begin{array}{c} \ln\;{SA}_{\text{pos}}\; \end{array}$	8310	1.143	1.628	0	8.475
lnRC	8310	1.265	1.728	0	8.569
struc	8310	0.393	0.135	0.014	0.890
lnpcGDP	8310	10.336	0.707	8.427	12.357
lnUrbPCD	8310	10.135	0.386	8.450	11.002
lnTSFAI	8310	13.681	1.152	8.912	17.772
lnRPop	8310	3.318	0.921	1.085	5.373
lnRSST	8310	7.001	1.003	4.143	9.186
lnSecInd	8310	12.669	1.476	8.119	16.274
lnTertIE	8310	1.543	1.242	−2.529	4.288
lnGPBE	8310	12.280	0.662	9.816	15.176
lnAc	8310	3.323	1.631	0	8.272

*Notes:* To mitigate the influence of extreme values, most variables in this study are incorporated into the models in logged form. Specifically, the core dependent variables, including the total number of reviews (RC), and the number of positive sentiment reviews ($\begin{array}{c} {SA}_{\text{pos}}\; \end{array}$), as well as other variables, are log-transformed before estimation, with the exception of the industrial structure (Struc).

## Results

### Decoding multisensory and ecocultural experiences through UGC analytics

**Fig 3:**
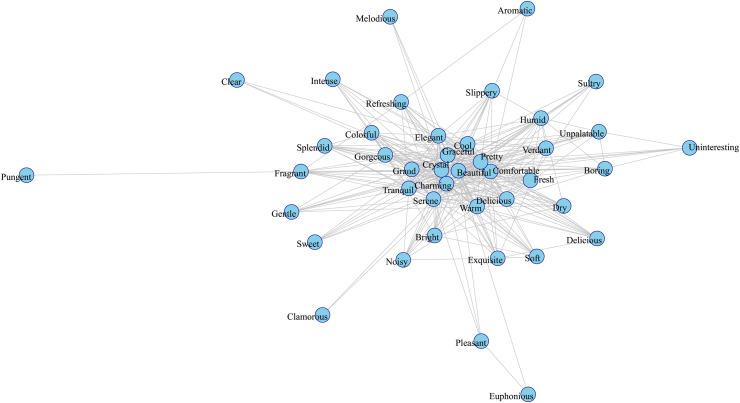
Cooccurrence Network of the Five Sensory Experiences.

**Fig 4:**
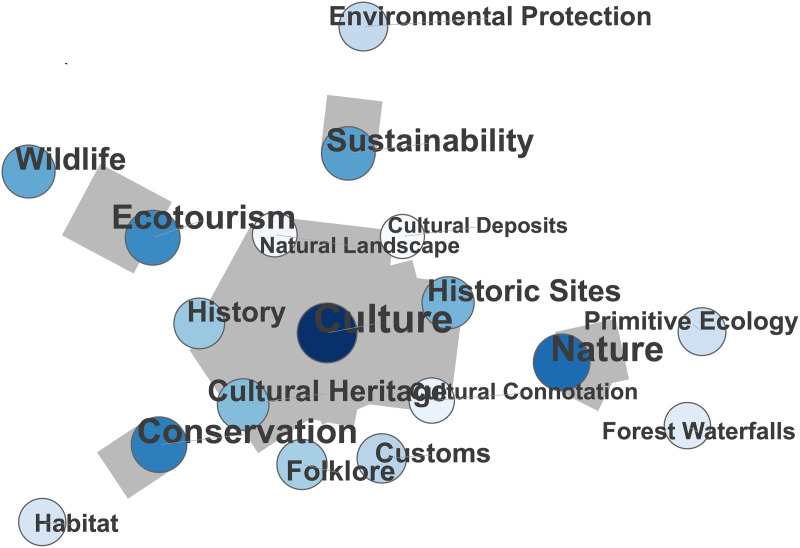
Cooccurrence Network of Ecological and Cultural Experiences.

Building upon sense-of-place ecology theory [6], this study advances the analytical framework for CESs in nature-based tourism by investigating multisensory experiential dimensions (“five sensory perceptions”) and place-based cultural cognition (“cultural perceptions”). Extending Ghermandi et al.’s cluster-based analysis of geotagged images [48], we applied 334,234 UGC entries from Dianping to reveal how sensory-cognitive hierarchies and ecocultural value networks collectively shape destination competitiveness. The resulting co-occurrence graphs for the “Five Sensory Experiences” and “Ecocultural Experiences” are shown in the co-occurrence network graphs (Fig 3, Fig 4).

In Fig 3, the high frequency and strong co-occurrence of keywords such as “charming” and “beautiful,” derived from the visual dimension, indicate that visual experiences dominate visitors’ perceptions. This observation aligns with the multisensory experience framework in tourism research [87], underscoring visual perception’s critical role in shaping visitor evaluations of scenic attractions and its influence on destination selection. The environmental contact dimension, which reflects visitors’ direct interactions with natural conditions such as temperature, humidity, and comfort, is indicated by the frequent appearance of keywords such as “comfortable,” “cool,” and “warm.” This result indicates tourists’ sensitivity to environmental comfort, highlighting the importance of visual and tactile impressions in tourism experiences [6]. Moreover, the vocabulary represented in the co-occurrence network captures the interplay of multisensory experiences. Olfactory impressions such as “aromatic” and “pungent” add a distinctive sensory layer, and auditory impressions such as “melodious” contribute to the ambiance, while gustatory terms imply diverse taste-related experiences. The multisensory integration of these inputs yields significant market potential in tourism. However, in addition to positive terms, such as “graceful” and “comfortable,” negative feedback, such as “boring,” “uninteresting,” and “unpalatable,” also emerges within the network. Analyzing such negative feedback enables destinations to identify areas of visitor dissatisfaction, thereby optimizing service delivery and mitigating adverse effects. For example, negative perceptions related to dining (“unpalatable”) and scenic attractiveness (“unattractive”) have a detrimental impact on the overall visitor experience, indicating potential areas for improvement.

Fig 4 shows that “nature,” “conservation,” and “culture” are key points in the network, highlighting how important natural landscapes are for shaping stories about culture and enhancing the experiences of visitors. Natural features, such as waterfalls and forests, function as multisensory repositories of biocultural value, offering aesthetic engagement while activating parallel cognitive processes of environmental stewardship and place-based cultural resonance among visitors. Interestingly, phrases such as “sustainability,” “environmental protection,” and “wildlife” suggest that tourists are concurrently learning about culture and ecology. These immersive experiences encourage greater knowledge and appreciation for ecosystems while reinforcing a sense of environmental responsibility. The spread of these ecological and cultural values helps create a more responsible tourist paradigm in which experiencing the beauty of nature simultaneously fosters awareness of environmental conservation.

### Benchmark regression results and analysis

Table 2 shows the impact of establishing NPs on improving nature-based tourism experiences. Column (1) demonstrates the ability of the establishment of NPs to drive experiential quality (β=0.528 , p<0.01 ). This finding suggests that the construction of NPs has a significantly positive effect on reviews expressing positive sentiment, increasing the relative level of positive nature-based tourism experiences by approximately $69.554\%(\approx \left(e^{0.528}-1\right)\times 100\%\backslash )$. In other words, the construction of NPs has positive multilevel effects that substantially enhance the tourism experience at natural scenic spots and increase visitors’ positive feedback. Consequently, picturesque places gain a better reputation, and travelers are more inclined to suggest these locations to others, thereby increasing the likelihood that those people will actually visit. Column (2) demonstrates a statistically significant coefficient (β=0.509 , p<0.01 ) for the Treated*post interaction term in explaining lnRC. The establishment of NPs increases destination engagement and market recognition, which are operationalized as $66.363\%(\approx \left(e^{0.509}-1\right)\times 100\%\backslash )$ increases in visitor-generated interaction intensity. Thus, the results confirm Hypothesis H1, which states that establishing NPs greatly improves the tourist experience in natural scenic regions. With the establishment of NPs, overall visitor input increases, site popularity increases, and positive feedback is generated, thereby enhancing the destination’s reputation.

**Table 2:** DID Estimation Results.

	Dependent Variable	
Variable	(1)	(2)
	$\begin{array}{c} \ln\;{SA}_{pos} \end{array}$	lnRC
Treated*post	0.528***	0.509***
	(0.174)	(0.179)
Control	Yes	Yes
Scenic Spot Fixed Effects	Yes	Yes
Time Fixed Effects	Yes	Yes
N	8,310	8,310
R-squared	0.522	0.541

*** *p* > 0.01, ** *p* > 0.05, * *p* > 0.1. Standard errors in parentheses.

SEs are clustered at the county level.

### Robustness checks

**Fig 5:**
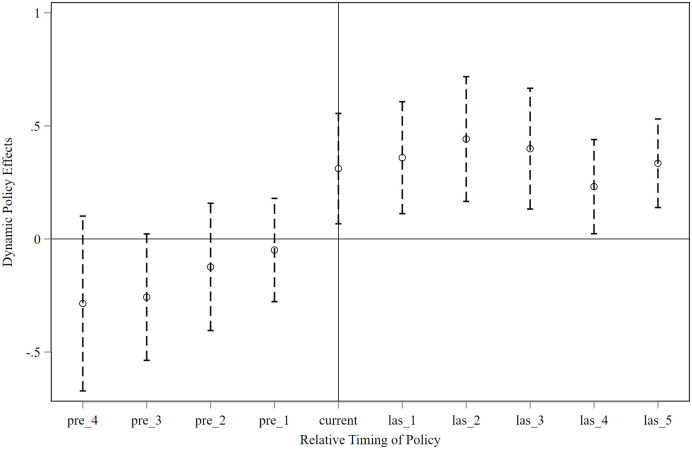
Parallel Trend Test for the Impact of the Construction of NPs on Nature-based Tourism Experiences.

According to the benchmark regression, the construction of NPs improved the experience of nature-based tourism more than other destinations located in ONP counties did. However, it is possible that such differences existed before the NPs were established. To address this concern, the study conducted a parallel trends test using an event-study approach. The results (Fig 5) show that the estimated coefficients are statistically insignificant and fluctuate around zero before NP construction, whereas the positive effects on nature-based tourism experiences become significant after construction began. Overall, the parallel trends assumption is satisfied by the model.

This study further conducted a series of robustness checks by adjusting the model specifications and excluding special samples. First, we implement a PSM-DID approach in which propensity scores are estimated using a logit model based on pre-treatment characteristics, followed by nearest-neighbor matching. The results indicate that under a more stringent matching design, the estimated coefficients remain consistent in sign, suggesting that the main conclusions exhibit a certain degree of robustness across alternative identification strategies. Second, to ensure that the results are not driven by the exceptional characteristics of natural and cultural heritage sites, we conducted an empirical analysis after eliminating all the observations from Jiuzhaigou County and Dujiangyan city, both of which are home to World Heritage sites. The findings confirm that the positive effect of the construction of NPs remains robust. For more information, see S2 and S3 Tables.

### Mechanism analysis

#### Impact of the fiscal expenditure mechanism.

The general public budget expenditure (GPBE) is directed primarily toward local developmental priorities and to ensure the effective provision of public services, including infrastructure and environmental quality. The estimated impact of NP development on fiscal expenditure is shown in Column (1) of Table 3. The estimated coefficient of treated*post is 0.082 and is statistically significant at the 5% level. This finding indicates that the construction of NPs significantly increases fiscal expenditure by approximately $8.546\%(\approx \left(e^{0.082}-1\right)\times 100\%\backslash )$. Existing studies indicate that fiscal expenditure constitutes a critical foundation for improving the quality and sustainability of public services at protected area tourism destinations [88]. In particular, enhancing the effective provision of public goods and facilities is identified as a key factor in meeting visitors‘ expectations regarding their tourism experiences [78]. This finding provides support for H2, which holds that the construction of NPs enhances nature-based tourism experiences by having a favorable impact on government expenditure.

**Table 3:** Results of the Mechanism Effect Test.

	Dependent Variable	
Variable	(1)	(2)
	lnGPBE	lnAC
Treated*post	0.082**	0.224**
	(0.038)	(0.095)
Control	Yes	Yes
Scenic Spot Fixed Effects	Yes	Yes
Time Fixed Effects	Yes	Yes
N	8310	8310
R-squared	0.823	0.895

*** p<0.01 , ** p<0.05 , * p<0.1 . Standard errors in parentheses.

SEs are clustered at the county level.

#### Impact of tourism industry scale mechanism.

This study measured the “lodging” scale of the tourism experience economy by measuring how many places, such as hotels and guesthouses, are available. This variable, which served as a proxy for the tourism industry scale, was used to investigate whether the construction of NPs improves nature-based tourism experiences by promoting the growth of the tourism industry scale. The estimated impact of the construction of NPs on the tourism industry scale is shown in column (2) of Table 3. The estimated coefficient of treated*post is 0.224 and is statistically significant at the 5% level. This result indicates that the construction of NPs significantly increases the tourism industry scale by approximately $25.107\%(\approx \left(e^{0.224}-1\right)\times 100\%\backslash )$. According to tourism experience theory, the supportive dimension of the tourism experience, composed of basic services such as accommodation and catering, constitutes a fundamental determinant of visitors‘ overall experiences [79]. This suggests that the expansion of the tourism industry scale, as reflected by accommodation capacity, effectively enhances the quality of tourists‘ functional experiences beyond main attractions. This synergy reflects an enhanced capacity for tourism-supporting resources, enabling improved service provision and greater diversification of visitor experiences. A growing tourism sector also facilitates the dissemination of destination reputation through social media platforms, generating a positive feedback loop that supports sustainable regional tourism development. Taken together, these findings offer robust empirical support for Hypothesis H3.

## Further analysis

**Fig 6:**
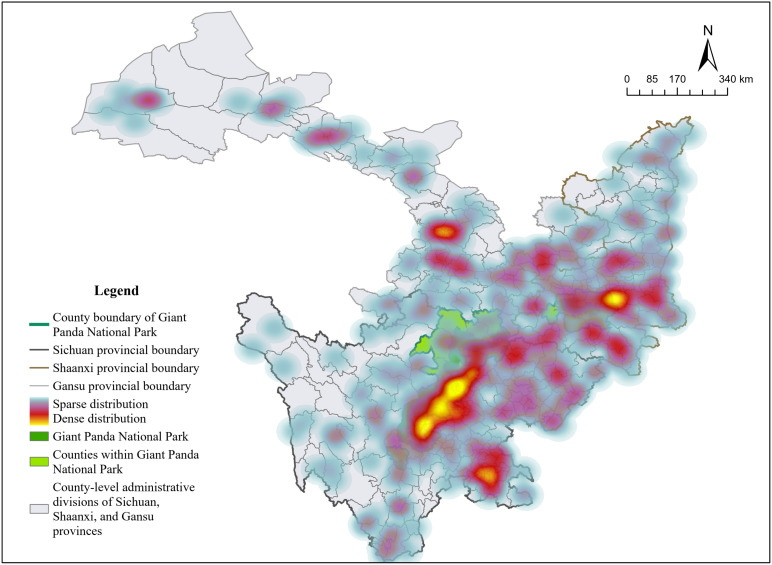
Spatial Distribution of Nature-based Tourism Experiences.

To examine the geospatial distribution patterns of the effects of establishing NPs on enhancing nature-based tourism, this study utilized geospatial datasets and GIS software to produce a spatial visualization of gradients in perceptions of tourism experience across areas of NP construction (Fig 6). Centering on the administrative boundaries of Sichuan, Shaanxi, and Gansu Provinces, the visualization delineates the distribution of tourism experiences across nature-based scenic areas of Grade A. This study offers insights into the intensity and spatial heterogeneity of visitor perceptions, thus providing a valuable reference for understanding the regional impacts of constructing NPs on nature-based tourism experiences.

The output of the geospatial analysis (Fig 6) reveals marked geospatial heterogeneity in enhancing nature-based tourism experiences. The analysis reveals a spatially clustered pattern in the enhancement of nature-based tourism experiences, driven by NP development. High-intensity clusters (highlighted in yellow-toned gradients) are concentrated in four key areas: (1) the southwestern Sichuan corridor of counties within GPNP, (2) the ecological tourism belt of Yibin, Sichuan Province, (3) the peripark development zone of Lanzhou, Gansu Province, and (4) the core tourism area of Chang’an District, Shaanxi Province.

To further examine the heterogeneity in the effects of the construction of NPs, this paper conducted an analysis with respect to the dimensions of regional differences and the intensity of policy impacts. First, we divided the sample into Sichuan, Shaanxi, and Gansu to assess regional heterogeneity based on provincial differences. We stratified the treatment group into the high-intensity policy group and the low-intensity policy group by the percentage of the land area of the WNP designated as NPs, as defined in the Pilot Plan for the Giant Panda National Park System [89], with the cutoff set at the sample mean, to test for differential policy intensity effects. Based on the full sample, we estimate two separate regressions. In one regression, observations belonging to the low-intensity policy group are excluded, while in the other regression, observations belonging to the high-intensity policy group are excluded. The regression results are presented in Table 4. At the regional level, the coefficients for the impact of the construction of GPNP on nature-based tourism experience are 0.454 in Sichuan and 0.538 in Shaanxi, both of which are statistically significant at the 5% level, while the result for Gansu is insignificant. This finding indicates that the policy has a stronger promotional effect on nature-based tourism in Shaanxi than in Sichuan, with no significant effect observed in Gansu. Compared with the spatial distribution of nature-based tourism capacity shown in Fig 6, these empirical results reveal the differential marginal effects of NP establishment across regions. Compared with Sichuan, Shaanxi, which started with a relatively weaker tourism base, gained more from the exogenous boost that the construction of NPs offered through improved infrastructure and brand building, which resulted in larger marginal gains. With respect to Gansu, geographical and supporting factors may have hindered the translation of policy into statistically observable effects. At the policy intensity level, the establishment of NPs has a significantly positive coefficient of 0.554 for the low-intensity policy group, which is statistically significant at the 1% level, whereas the coefficient for the high-intensity policy group is insignificant. This finding suggests that the policy has a more substantial effect on enhancing nature-based tourism experiences in areas with lower policy intensity than in those with higher policy intensity. This outcome may be explained by the stricter regulatory constraints imposed on the high-intensity policy group. Within GPNP, land is zoned into core protection areas, where human activities are generally prohibited, and general control areas, where limited activities are permitted only under strict ecological management. As a result, for counties in the high-intensity policy group, where the designated NP area accounts for more than 32% of the county’s total area, the GPNP policy has no statistically significant effect on the nature-based tourism experience.

**Table 4:** Results of the heterogeneity analysis.

	Region			Policy Intensity	
	(1)	(2)	(3)	(4)	(5)
	Sichuan	Shaanxi	Gansu	High-intensity Policy Group	Low-intensity Policy Group
Treated*post	0.454**	0.538**	0.193	0.428	0.554**
	(0.225)	(0.219)	(0.394)	(0.269)	(0.208)
Control	Yes	Yes	Yes	Yes	Yes
Scenic Spot Fixed Effects	Yes	Yes	Yes	Yes	Yes
Time Fixed Effects	Yes	Yes	Yes	Yes	Yes
N	4335	2190	1785	7170	7875
R-squared	0.531	0.556	0.483	0.511	0.518

$\backslash {(}^{***}p<0.01\;$, $\backslash {(}^{**}p<0.05\;$, $\backslash {(}^{*}p<0.1\;$. Standard errors in parentheses.

SEs are clustered at the county level.

## Conclusions and recommendations

### Conclusions

In this study, GPNP was taken as a representative case to examine the effects of China’s NP construction on nature-based tourism experiences. The analysis covered the period from 2009 to 2023 and applied a DID framework to identify the causal effects of the policy. The primary analytical units are Grade A nature-based tourist destinations that have been approved by the Sichuan, Shaanxi, and Gansu Culture and Tourism Administrations. Among them, 100 attractions located within the counties included in GPNP are classified as the treatment group, while 454 attractions located outside these counties serve as the control group. By evaluating the impact of NPs on nature-based tourism experiences, this research aims to generate empirical evidence and actionable insights that can inform the planning and management of other wildlife-oriented NPs in China and beyond. Studies indicate that in nature-based tourism, visual, tactile, and olfactory stimuli influence visitors’ assessments of their experiences. Among these senses, the co-occurrence network analysis (Fig 3) reveals that visual and tactile descriptors occupy central positions within the network. However, olfactory perceptions are not an independent sensory modality; rather, they interact with other sensory channels and are closely integrated into the overall sensory experience. Furthermore, empirical analysis shows that the construction of NPs leads to multilevel impacts, resulting in improved nature-based tourism experiences in designated counties. These improvements are correlated with enhanced perceptions of the quality of the tourism experience, thereby supporting the role of NPs in enhancing the experiential value of nature-based tourism.

Mechanistic analysis suggests that improvements in nature-based tourism experiences associated with NP development may operate through two potential channels: the expansion of the tourism industry scale and increases in fiscal expenditure. Empirical results indicate that NP construction has a statistically significant effect on both tourism industry scale and fiscal expenditure, which, when interpreted in light of existing studies and relevant theory, provides evidence that these two factors constitute key pathways through which NP construction enhances nature-based tourism experiences.

To examine the spatial effects of constructing NPs on nature-based tourism experiences, this study conducted a visualization analysis of review data. The heatmap distributions reveal a clear spatial clustering pattern in the impacts of NP development. Heterogeneity analyses show pronounced regional and policy-intensity differences in the policy’s effects. At the regional level, significant positive impacts are observed in Shaanxi and Sichuan, with the effect in Shaanxi exceeding that in Sichuan, while no significant effect is detected in Gansu. These results suggest that differences in initial conditions, particularly in resource endowments and infrastructure, may be underlying factors contributing to the regional variation in policy effects. With respect to the policy intensity, the results show that while the NP policy has a statistically insignificant effect on counties in the high-intensity policy group, it has a significant positive effect on counties in the low-intensity policy group. This difference may be attributable to the stricter management constraints faced by the high-intensity policy group.

### Recommendations

Based on the findings from GPNP, this study provides evidence-based recommendations for establishing and managing protected areas with a primary focus on wildlife conservation. Three operational recommendations emerge from the evidence-based conclusions about the impacts of the NP policy on nature tourism experiences.

First, data-driven social media analytics frameworks should be implemented to facilitate bidirectional value cocreation between NPs and visitors. Nature-based tourism in NPs should prioritize visitors’ diverse and evolving needs, particularly in enhancing their tourism experiences. This data-driven approach facilitates precision enhancements in tourism service delivery through systematic analysis of experiential feedback. For example, the comprehensive enhancement of visitors’ multisensory experiences can be achieved by preserving the integrity and aesthetic appeal of natural landscapes, enhancing their botanical scents, creating tactile interaction zones with natural elements, and adapting facilities to accommodate extreme weather conditions. Such data-driven optimizations can meet tourist demands and amplify destination visibility through user-generated social media dissemination. By employing social media analytics for collaborative value creation, destinations can achieve continuous service refinement and strengthened visitor engagement, with mutually beneficial outcomes.

Second, strengthening the integration between NPs and service-oriented industries can support the cultivation of development models that balance ecological integrity and economic production. By leveraging the natural resource advantages of NPs, nature-dependent service sectors can be innovatively reconfigured to establish a symbiotic equilibrium between conservation imperatives and commercial viability. This synergy empirically illustrates the simultaneous optimization of the quality of nature-based tourist experiences and localized sustainable economic changes. Within culinary services, the strategic integration of indigenous natural and cultural assets enables the creation of terroir-specific gourmet experiences that improve the gustatory component of tourism. Simultaneously, accommodation operators can use the ecological capital of NPs to develop ecofriendly lodging alternatives that correspond with environmentally conscious consumer desires. These sector-specific innovations work together to make nature-based locations more appealing.

Third, in the context of the interprovincial differences in the tourism experiences generated by NP establishment, the collaborative efforts of Sichuan, Shaanxi, and Gansu should focus on targeted, context-specific policies rather than standardized measures. Specifically, the three provinces should coordinate according to their respective stages of development: Shaanxi and Sichuan, where the policy effects are pronounced, can share successful management and promotion experiences, while Gansu, where the effects are less evident, should receive more tailored capacity-building and resource allocation within the collaborative framework to translate its ecological potential into tourism attractiveness. Policy implementation should also be adapted to local conditions: in areas with strict management and heavy restrictions, more flexible approaches are needed to enhance visitor experiences; in relatively lenient areas, efforts should focus on consolidating existing gains and preventing overdevelopment. By recognizing differences and leveraging complementary advantages, this collaborative approach can effectively narrow regional gaps, ensuring that national park development in these three provinces promotes nature-based tourism experiences in park counties on an ecologically sustainable basis, thus offering visitors high-quality and distinctive experiences.

## Supporting information

S1 Table DID estimation results with all control variables. (DOCX)

S2 Table PSM-DID robustness check results with all control variables. (DOCX)

S3 Table Robustness check excluding special samples. (DOCX)

S4 Table Results of mechanism effect test with all control variables. (DOCX)

S5 Table Heterogeneity analysis results with all control variables. (DOCX)

S6 Table County-level matched sample list and weights. (DOCX)

S1 Fig Post-matching covariate balance check. (TIF)

S2 Fig Pre-matching kernel density plot. (TIF)

S3 Fig Post-matching kernel density plot. (TIF)

S4 Fig Placebo test. (TIF)
