# Supplementary material for: Has the establishment of national parks improved nature-based tourism experiences? Evidence from social media data
Source: PLoS One. 2026 Mar 20;21(3):e0343256. doi: 10.1371/journal.pone.0343256 (PMC13004529; doi:10.1371/journal.pone.0343256)
Supplement: S1 Table — (DOCX) [file pone.0343256.s001.docx]

S1 Table. DID Estimation Results with All Control Variables

|  | (1) | (2) |
| --- | --- | --- |
|  | lnSApos | lnRC |
| Treated*post | 0.528*** | 0.509*** |
|  | (0.174) | (0.179) |
| struc | 0.617 | 0.659 |
|  | (0.439) | (0.445) |
| lnpcGDP | -0.117 | -0.108 |
|  | (0.135) | (0.139) |
| lnUrbPCDI | -0.341* | -0.327 |
|  | (0.196) | (0.206) |
| lnTSFAI | -0.058 | -0.068 |
|  | (0.042) | (0.043) |
| lnRPop | 0.591*** | 0.600*** |
|  | (0.195) | (0.199) |
| lnRSST | -0.063 | -0.081 |
|  | (0.076) | (0.081) |
| lnSecInd | 0.022 | 0.031 |
|  | (0.071) | (0.073) |
| lnTertIE | 0.017 | 0.014 |
|  | (0.028) | (0.029) |
| Scenic Spot Fixed Effects | YES | YES |
| Time Fixed Effects | YES | YES |
| N | 8310 | 8310 |
| R-squared | 0.522 | 0.541 |

*** p<0.01, ** p<0.05, * p<0.1 Robust standard errors in parentheses. SEs are clustered at the county level.
