# Supplementary material for: Has the establishment of national parks improved nature-based tourism experiences? Evidence from social media data
Source: PLoS One. 2026 Mar 20;21(3):e0343256. doi: 10.1371/journal.pone.0343256 (PMC13004529; doi:10.1371/journal.pone.0343256)
Supplement: S2 Table — (DOCX) [file pone.0343256.s002.docx]

S2 Table. PSM-DID Robustness Check Results with All Control Variables

|  | (1) | (2) |
| --- | --- | --- |
|  | lnSA_Pos | lnRC |
| Treated*post | 0.396* | 0.367* |
|  | (0.202) | (0.209) |
| struc | 0.416 | 0.372 |
|  | (0.799) | (0.799) |
| lnpcGDP | -0.560* | -0.574* |
|  | (0.288) | (0.298) |
| lnUrbPCDI | 0.109 | 0.111 |
|  | (0.517) | (0.566) |
| lnTSFAI | -0.049 | -0.055 |
|  | (0.076) | (0.079) |
| lnRPop | 0.466 | 0.514 |
|  | (0.317) | (0.343) |
| lnRSST | -0.102 | -0.096 |
|  | (0.115) | (0.114) |
| lnSecInd | 0.105 | 0.130 |
|  | (0.087) | (0.089) |
| lnTertIE | -0.008 | -0.020 |
|  | (0.048) | (0.050) |
| Scenic Spot Fixed Effects | Yes | Yes |
| Time Fixed Effects | Yes | Yes |
| N | 2133 | 2133 |
| R-squared | 0.579 | 0.590 |

*** p<0.01, ** p<0.05, * p<0.1 Robust standard errors in parentheses. SEs are clustered at the county level.
