# Supplementary material for: Has the establishment of national parks improved nature-based tourism experiences? Evidence from social media data
Source: PLoS One. 2026 Mar 20;21(3):e0343256. doi: 10.1371/journal.pone.0343256 (PMC13004529; doi:10.1371/journal.pone.0343256)
Supplement: S3 Table — (DOCX) [file pone.0343256.s003.docx]

S3 Table. Robustness Check Excluding Special Samples

|  | Excluding Jiuzhaigou County | | Excluding Dujiangyan City | |
| --- | --- | --- | --- | --- |
|  | lnSApos | lnRC | lnSApos | lnRC |
| Treated*post | 0.523*** | 0.509*** | 0.496*** | 0.475*** |
|  | (0.178) | (0.182) | (0.178) | (0.183) |
| struc | 0.621 | 0.655 | 0.660 | 0.703 |
|  | (0.440) | (0.446) | (0.439) | (0.445) |
| lnpcGDP | -0.114 | -0.106 | -0.109 | -0.100 |
|  | (0.135) | (0.139) | (0.134) | (0.138) |
| lnUrbPCDI | -0.342* | -0.328 | -0.378* | -0.366* |
|  | (0.196) | (0.206) | (0.197) | (0.206) |
| lnTSFAI | -0.058 | -0.067 | -0.055 | -0.065 |
|  | (0.042) | (0.043) | (0.042) | (0.043) |
| lnRPop | 0.592*** | 0.600*** | 0.580*** | 0.589*** |
|  | (0.195) | (0.199) | (0.195) | (0.199) |
| lnRSST | -0.063 | -0.081 | -0.061 | -0.079 |
|  | (0.076) | (0.081) | (0.075) | (0.080) |
| lnSecInd | 0.023 | 0.030 | 0.022 | 0.031 |
|  | (0.072) | (0.073) | (0.072) | (0.073) |
| lnTertIE | 0.018 | 0.014 | 0.018 | 0.015 |
|  | (0.028) | (0.029) | (0.028) | (0.029) |
| Scenic Spot Fixed Effects | YES | YES | YES | YES |
| Time Fixed Effects | YES | YES | YES | YES |
| N | 8280 | 8280 | 8250 | 8250 |
| R-squared | 0.521 | 0.541 | 0.520 | 0.540 |

*** p<0.01, ** p<0.05, * p<0.1 Robust standard errors in parentheses. SEs are clustered at the county level.
