# Supplementary material for: Has the establishment of national parks improved nature-based tourism experiences? Evidence from social media data
Source: PLoS One. 2026 Mar 20;21(3):e0343256. doi: 10.1371/journal.pone.0343256 (PMC13004529; doi:10.1371/journal.pone.0343256)
Supplement: S4 Table — (DOCX) [file pone.0343256.s004.docx]

S4 Table. Results of Mechanism Effect Test with All Control Variables

|  | (1) | (2) |
| --- | --- | --- |
|  | lnGPBE | lnAC |
| Treated*post | 0.082** | 0.224** |
|  | (0.038) | (0.095) |
| struc | 0.110 | -0.007 |
|  | (0.135) | (0.270) |
| lnpcGDP | -0.002 | -0.044 |
|  | (0.040) | (0.241) |
| lnUrbPCDI | -0.176*** | -0.168 |
|  | (0.052) | (0.156) |
| lnTSFAI | 0.075*** | -0.003 |
|  | (0.012) | (0.040) |
| lnRPop | 0.165*** | 0.336** |
|  | (0.060) | (0.154) |
| lnRSST | 0.007 | 0.165*** |
|  | (0.024) | (0.047) |
| lnSecInd | 0.030 | 0.025 |
|  | (0.026) | (0.075) |
| lnTertIE | 0.004 | 0.010 |
|  | (0.007) | (0.021) |
| Scenic Spot Fixed Effects | YES | YES |
| Time Fixed Effects | YES | YES |
| N | 8310 | 8310 |
| R-squared | 0.823 | 0.895 |

*** p<0.01, ** p<0.05, * p<0.1 Robust standard errors in parentheses. SEs are clustered at the county level.
