# Supplementary material for: Has the establishment of national parks improved nature-based tourism experiences? Evidence from social media data
Source: PLoS One. 2026 Mar 20;21(3):e0343256. doi: 10.1371/journal.pone.0343256 (PMC13004529; doi:10.1371/journal.pone.0343256)
Supplement: S5 Table — (DOCX) [file pone.0343256.s005.docx]

S5 Table. Heterogeneity Analysis Results with All Control Variables

|  | (1) | (2) | (3) | (4) | (5) |
| --- | --- | --- | --- | --- | --- |
|  | Sichuan | Shaanxi | Gansu | High Policy  Impact Group | Low Policy  Impact Group |
| Treated*post | 0.454** | 0.538** | 0.193 | 0.428 | 0.554*** |
|  | (0.225) | (0.219) | (0.394) | (0.269) | (0.208) |
| struc | 0.375 | 1.687*** | -0.493 | 0.626 | 0.595 |
|  | (1.075) | (0.561) | (0.641) | (0.469) | (0.446) |
| lnpcGDP | 0.441 | -0.112 | -0.490*** | -0.031 | -0.074 |
|  | (0.322) | (0.230) | (0.174) | (0.138) | (0.135) |
| lnUrbPCDI | -0.407 | 0.111 | -0.033 | -0.444** | -0.337* |
|  | (0.347) | (0.492) | (0.234) | (0.200) | (0.198) |
| lnTSFAI | -0.113* | -0.090 | -0.038 | -0.055 | -0.050 |
|  | (0.067) | (0.100) | (0.058) | (0.045) | (0.042) |
| lnRPop | 1.398*** | 0.347* | 0.283 | 0.645*** | 0.539*** |
|  | (0.495) | (0.183) | (0.555) | (0.214) | (0.193) |
| lnRSST | -0.173 | 0.047 | -0.304 | -0.037 | -0.060 |
|  | (0.143) | (0.085) | (0.298) | (0.072) | (0.077) |
| lnSecInd | -0.141 | -0.012 | -0.163 | -0.053 | 0.029 |
|  | (0.218) | (0.089) | (0.137) | (0.085) | (0.072) |
| lnTertIE | 0.025 | 0.069 | -0.056 | 0.021 | 0.024 |
|  | (0.034) | (0.054) | (0.042) | (0.028) | (0.029) |
| Scenic Spot Fixed Effects | YES | YES | YES | YES | YES |
| Time Fixed Effects | YES | YES | YES | YES | YES |
| N | 4335 | 2190 | 1785 | 7170 | 7875 |
| R-squared | 0.531 | 0.556 | 0.483 | 0.511 | 0.518 |

*** p<0.01, ** p<0.05, * p<0.1 Robust standard errors in parentheses. SEs are clustered at the county level.
