# Supplementary material for: Has the establishment of national parks improved nature-based tourism experiences? Evidence from social media data
Source: PLoS One. 2026 Mar 20;21(3):e0343256. doi: 10.1371/journal.pone.0343256 (PMC13004529; doi:10.1371/journal.pone.0343256)
Supplement: S6 Table — (DOCX) [file pone.0343256.s006.docx]

S6 Table. County-level Matched Sample List and Weights

| Prov | City | County | Within Giant Panda National Park (Yes/No) | spot_count | County_weight |
| --- | --- | --- | --- | --- | --- |
| Sichuan Province | Aba Prefecture | Jiuzhaigou | Yes | 2 | 0.36101083 |
|  | Deyang | Shifang City | Yes | 3 | 0.541516245 |
|  | Mianyang | Beichuan | Yes | 5 | 0.902527076 |
|  | Chengdu | Dayi | Yes | 4 | 0.722021661 |
|  | Ya'an | Tianquan | Yes | 2 | 0.36101083 |
|  | Mianyang | Anzhou | Yes | 4 | 0.722021661 |
|  | Ya'an | Baoxing | Yes | 5 | 0.902527076 |
|  | Chengdu | Chongzhou | Yes | 4 | 0.722021661 |
|  | Mianyang | Pingwu | Yes | 6 | 1.083032491 |
|  | Chengdu | Pengzhou City | Yes | 4 | 0.722021661 |
|  | Aba Prefecture | Songpan | Yes | 3 | 0.541516245 |
|  | Aba Prefecture | Wenchuan | Yes | 2 | 0.36101083 |
|  | Meishan | Hongya | Yes | 2 | 0.36101083 |
|  | Ya'an | Shimian | Yes | 3 | 0.541516245 |
|  | Deyang | Mianzu | Yes | 5 | 0.902527076 |
|  | Ya'an | Lushan | Yes | 4 | 0.722021661 |
|  | Aba Prefecture | Maoxian | Yes | 4 | 0.722021661 |
|  | Ya'an | Yingjing | Yes | 3 | 0.541516245 |
|  | Chengdu | Dujiangyan City | Yes | 4 | 0.722021661 |
|  | Guangyuan | Qingchuan | Yes | 5 | 0.902527076 |
| Shaanxi Province | Hanzhong | Foping | Yes | 2 | 0.36101083 |
|  | Xi'an | Zhouzhi | Yes | 4 | 0.722021661 |
|  | Baoji | Taibai | Yes | 5 | 0.902527076 |
|  | Ankang | Ningshan | Yes | 1 | 0.180505415 |
|  | Hanzhong | Yangxian | Yes | 3 | 0.541516245 |
|  | Hanzhong | Liuba | Yes | 1 | 0.180505415 |
|  | Baoji | Meixian | Yes | 2 | 0.36101083 |
|  | Xi'an | Huyi District | Yes | 4 | 0.722021661 |
| Gansu Province | Longnan | Wenxian | Yes | 3 | 0.541516245 |
|  | Longnan | Wudu District | Yes | 1 | 0.180505415 |
| Sichuan Province | Dazhou | Wanyuan | No | 2 | 0.36101083 |
|  | Neijiang | Dongxing District | No | 1 | 0.180505415 |
|  | Ganzi Prefecture | Danba | No | 1 | 0.180505415 |
|  | Meishan | Danling | No | 1 | 0.180505415 |
|  | Leshan | Shizhong District, Leshan City | No | 1 | 0.180505415 |
|  | Ganzi Prefecture | Jiulong | No | 1 | 0.180505415 |
|  | Ganzi Prefecture | Xiangcheng | No | 1 | 0.180505415 |
|  | Panzhihua | Renhe | No | 2 | 0.36101083 |
|  | Meishan | Renshou | No | 1 | 0.180505415 |
|  | Nanchong | Yilong | No | 1 | 0.180505415 |
|  | Liangshan Prefecture | Huidong | No | 1 | 0.180505415 |
|  | Liangshan Prefecture | Huili | No | 2 | 0.36101083 |
|  | Yibin | Xinghui | No | 4 | 0.722021661 |
|  | Liangshan Prefecture | Mianning | No | 2 | 0.36101083 |
|  | Guangyuan | Lizhou District | No | 2 | 0.36101083 |
|  | Guangyuan | Jiange | No | 2 | 0.36101083 |
|  | Guang'an | Huaying City | No | 1 | 0.180505415 |
|  | Bazhong | Nanjiang | No | 2 | 0.36101083 |
|  | Yibin | Nanxi District | No | 1 | 0.180505415 |
|  | Nanchong | Nanbu | No | 4 | 0.722021661 |
|  | Chengdu | Shuangliu District | No | 2 | 0.36101083 |
|  | Yibin | XUzhou District | No | 3 | 0.541516245 |
|  | Luzhou | Gulin | No | 2 | 0.36101083 |
|  | Luzhou | Hejiang | No | 1 | 0.180505415 |
|  | Ya'an | Mingshan District | No | 3 | 0.541516245 |
|  | Dazhou | Dazu | No | 2 | 0.36101083 |
|  | Suining | Daying | No | 2 | 0.36101083 |
|  | Neijiang | Weiyuan | No | 3 | 0.541516245 |
|  | Liangshan Prefecture | Ningnan | No | 1 | 0.180505415 |
|  | Dazhou | Xuanhan | No | 4 | 0.722021661 |
|  | Zigong | Fushun | No | 1 | 0.180505415 |
|  | Aba Prefecture | Xiaojin | No | 3 | 0.541516245 |
|  | Guang'an | Yueci | No | 2 | 0.36101083 |
|  | Leshan | Emeishan City | No | 1 | 0.180505415 |
|  | Leshan | Ebian | No | 1 | 0.180505415 |
|  | Ganzi Prefecture | Batang | No | 1 | 0.180505415 |
|  | Bazhong | Bazhou District | No | 2 | 0.36101083 |
|  | Bazhong | Pingchang | No | 6 | 1.083032491 |
|  | Deyang | Guanghan | No | 2 | 0.36101083 |
|  | Ganzi Prefecture | Kangding City | No | 2 | 0.36101083 |
|  | Meishan | Pengshan District | No | 1 | 0.180505415 |
|  | Ganzi Prefecture | Dege | No | 1 | 0.180505415 |
|  | Bazhong | Enyang District | No | 1 | 0.180505415 |
|  | Chengdu | Xinjin | No | 3 | 0.541516245 |
|  | Chengdu | Xindu | No | 1 | 0.180505415 |
|  | Deyang | Jingyang District | No | 2 | 0.36101083 |
|  | Guangyuan | Wangcang | No | 4 | 0.722021661 |
|  | Guangyuan | Zhaohua District | No | 5 | 0.902527076 |
|  | Liangshan Prefecture | Puge | No | 1 | 0.180505415 |
|  | Guangyuan | Chaotian District | No | 3 | 0.541516245 |
|  | Mianyang | Zitong | No | 2 | 0.36101083 |
|  | Ya'an | Hanyuan | No | 1 | 0.180505415 |
|  | Yibin | Jiangan | No | 1 | 0.180505415 |
|  | Mianyang | Jiangyou | No | 2 | 0.36101083 |
|  | Luzhou | Jiangyang District | No | 2 | 0.36101083 |
|  | Leshan | Muchuan | No | 1 | 0.180505415 |
|  | Zigong | Yantan | No | 1 | 0.180505415 |
|  | Luzhou | Luxian | No | 3 | 0.541516245 |
|  | Ganzi Prefecture | Luding | No | 2 | 0.36101083 |
|  | Dazhou | Quxian | No | 1 | 0.180505415 |
|  | Chengdu | Wenjiang District | No | 1 | 0.180505415 |
|  | Mianyang | Youxian District | No | 1 | 0.180505415 |
|  | Leshan | Jianwei | No | 2 | 0.36101083 |
|  | Yibin | Gongxian | No | 2 | 0.36101083 |
|  | Aba Prefecture | Lixian | No | 3 | 0.541516245 |
|  | Ganzi Prefecture | Litang | No | 3 | 0.541516245 |
|  | Mianyang | Yanting | No | 1 | 0.180505415 |
|  | Liangshan Prefecture | Yanyuan | No | 1 | 0.180505415 |
|  | Panzhihua | Yanbian | No | 3 | 0.541516245 |
|  | Ganzi Prefecture | ShiQu | No | 2 | 0.36101083 |
|  | Ganzi Prefecture | Daocheng | No | 2 | 0.36101083 |
|  | Chengdu | Jianyang | No | 1 | 0.180505415 |
|  | Panzhihua | Miyi | No | 2 | 0.36101083 |
|  | Luzhou | Naxi District | No | 2 | 0.36101083 |
|  | Yibin | Cuiping District | No | 3 | 0.541516245 |
|  | Zigong | Ziliujing District | No | 1 | 0.180505415 |
|  | Suining | Chuanshan District | No | 3 | 0.541516245 |
|  | Ganzi Prefecture | Seda | No | 1 | 0.180505415 |
|  | Aba Prefecture | Jorge | No | 4 | 0.722021661 |
|  | Nanchong | Peng'an | No | 2 | 0.36101083 |
|  | Suining | Pengxi | No | 1 | 0.180505415 |
|  | Liangshan Prefecture | Xichang City | No | 3 | 0.541516245 |
|  | Zigong | Gongjing District | No | 1 | 0.180505415 |
|  | Liangshan Prefecture | Yuexi | No | 1 | 0.180505415 |
|  | Dazhou | Dachuan District | No | 2 | 0.36101083 |
|  | Bazhong | Tongjiang | No | 2 | 0.36101083 |
|  | Ganzi Prefecture | Daofu | No | 5 | 0.902527076 |
|  | Chengdu | Qionglai Market | No | 4 | 0.722021661 |
|  | Guang'an | Linshui | No | 4 | 0.722021661 |
|  | Chengdu | Pidu District | No | 2 | 0.36101083 |
|  | Leshan | Chrysostom River District | No | 1 | 0.180505415 |
|  | Chengdu | Jintang | No | 2 | 0.36101083 |
|  | Aba Prefecture | Jinchuan | No | 2 | 0.36101083 |
|  | Chengdu | Jinjiang District | No | 1 | 0.180505415 |
|  | Yibin | Changning | No | 5 | 0.902527076 |
|  | Nanchong | Langzhong Market | No | 1 | 0.180505415 |
|  | Aba Prefecture | Aba | No | 2 | 0.36101083 |
|  | Neijiang | Longchang | No | 3 | 0.541516245 |
|  | Ziyang | Yanjiang District | No | 1 | 0.180505415 |
|  | Ganzi Prefecture | Yajiang | No | 1 | 0.180505415 |
|  | Ya'an | Yucheng District | No | 4 | 0.722021661 |
|  | Liangshan Prefecture | Leibo | No | 1 | 0.180505415 |
|  | Meishan | Qingshen | No | 1 | 0.180505415 |
|  | Nanchong | Shunqing District | No | 2 | 0.36101083 |
|  | Aba Prefecture | Malcon City | No | 1 | 0.180505415 |
|  | Yibin | Gao | No | 1 | 0.180505415 |
|  | Nanchong | Gaoping District | No | 1 | 0.180505415 |
|  | Aba Prefecture | Heishui | No | 2 | 0.36101083 |
|  | Chengdu | Longquanyi District | No | 3 | 0.541516245 |
| Shaanxi Province | Weinan | Linwei District | No | 1 | 0.180505415 |
|  | Shangluo | Danfeng | No | 2 | 0.36101083 |
|  | Yulin | Jiaxian | No | 3 | 0.541516245 |
|  | Baoji | Fengxian | No | 3 | 0.541516245 |
|  | Baoji | Fengxiang | No | 1 | 0.180505415 |
|  | Hanzhong | Mian | No | 1 | 0.180505415 |
|  | Weinan | Huazhou District | No | 1 | 0.180505415 |
|  | Weinan | Huayin City | No | 1 | 0.180505415 |
|  | Hanzhong | Nanzheng District | No | 3 | 0.541516245 |
|  | Tongchuan | Yintai District | No | 2 | 0.36101083 |
|  | Weinan | Heyang | No | 2 | 0.36101083 |
|  | Shangluo | Shangnan | No | 2 | 0.36101083 |
|  | Shangluo | Shangzhou District | No | 4 | 0.722021661 |
|  | Hanzhong | Chenggu | No | 1 | 0.180505415 |
|  | Yan'an | His son was Chang Shi | No | 1 | 0.180505415 |
|  | Hanzhong | Ningqiang | No | 1 | 0.180505415 |
|  | Yan'an | Ansai District | No | 1 | 0.180505415 |
|  | Tongchuan | Yijun | No | 1 | 0.180505415 |
|  | Yan'an | Yichuan | No | 2 | 0.36101083 |
|  | Yan'an | Pagoda area | No | 2 | 0.36101083 |
|  | Weinan | Fuping | No | 1 | 0.180505415 |
|  | Shangluo | Shanyang | No | 2 | 0.36101083 |
|  | Ankang | Langao | No | 3 | 0.541516245 |
|  | Ankang | Pingli | No | 2 | 0.36101083 |
|  | Yulin | Fugu | No | 1 | 0.180505415 |
|  | Yan'an | Yanchuan | No | 1 | 0.180505415 |
|  | Yan'an | Yanchang | No | 1 | 0.180505415 |
|  | Baoji | Fufeng | No | 1 | 0.180505415 |
|  | Shangluo | Zhashui | No | 4 | 0.722021661 |
|  | Yulin | Yuyang District | No | 4 | 0.722021661 |
|  | Xianyang | Yongshou | No | 1 | 0.180505415 |
|  | Hanzhong | Hantai District | No | 2 | 0.36101083 |
|  | Ankang | Hanbin District | No | 6 | 1.083032491 |
|  | Ankang | Hanyin | No | 2 | 0.36101083 |
|  | Xianyang | Jingyang | No | 1 | 0.180505415 |
|  | Shangluo | Luonan | No | 6 | 1.083032491 |
|  | Xianyang | Chunhua | No | 2 | 0.36101083 |
|  | Yulin | Qingjian | No | 3 | 0.541516245 |
|  | Baoji | Weibin District | No | 1 | 0.180505415 |
|  | Weinan | Tongguan | No | 2 | 0.36101083 |
|  | Weinan | Chengcheng | No | 1 | 0.180505415 |
|  | Xi'an | Baqiao District | No | 2 | 0.36101083 |
|  | Yan'an | Ganquan | No | 1 | 0.180505415 |
|  | Weinan | Baishui | No | 1 | 0.180505415 |
|  | Ankang | Baihe | No | 1 | 0.180505415 |
|  | Ankang | Shiquan | No | 7 | 1.263537906 |
|  | Yulin | Shenmu City | No | 2 | 0.36101083 |
|  | Tongchuan | Yaozhou District | No | 3 | 0.541516245 |
|  | Xi'an | Lantian | No | 4 | 0.722021661 |
|  | Hanzhong | Xixiang | No | 1 | 0.180505415 |
|  | Ankang | Zhenping | No | 2 | 0.36101083 |
|  | Shangluo | Zhen'an | No | 2 | 0.36101083 |
|  | Hanzhong | Zhenba | No | 1 | 0.180505415 |
|  | Xi'an | Chang'an District | No | 6 | 1.083032491 |
|  | Xianyang | Changwu | No | 1 | 0.180505415 |
|  | Baoji | Long | No | 3 | 0.541516245 |
|  | Baoji | Chencang District | No | 1 | 0.180505415 |
|  | Xi'an | Yanta District | No | 1 | 0.180505415 |
|  | Yulin | Jingbian | No | 1 | 0.180505415 |
|  | Yan'an | Huanglong | No | 2 | 0.36101083 |
| Gansu Province | Lanzhou | Qili River District | No | 2 | 0.36101083 |
|  | Longnan | Liangdang | No | 1 | 0.180505415 |
|  | Zhangye | Linze | No | 2 | 0.36101083 |
|  | Dingxi | Lintao | No | 1 | 0.180505415 |
|  | Gannan Prefecture | Lintan | No | 1 | 0.180505415 |
|  | Wuwei | Liangzhou District | No | 5 | 0.902527076 |
|  | Pingliang | Huating City | No | 1 | 0.180505415 |
|  | Qingyang | Huachi | No | 1 | 0.180505415 |
|  | Gannan Prefecture | Zhuoni | No | 1 | 0.180505415 |
|  | Qingyang | Heshui | No | 1 | 0.180505415 |
|  | Linxia Prefecture | Hezheng | No | 2 | 0.36101083 |
|  | Jiayuguan | Jiayuguan Market | No | 2 | 0.36101083 |
|  | Lanzhou | Chengguan District | No | 2 | 0.36101083 |
|  | Gannan Prefecture | Xiahe | No | 2 | 0.36101083 |
|  | Lanzhou | Anning District | No | 1 | 0.180505415 |
|  | Longnan | Dangchang | No | 1 | 0.180505415 |
|  | Zhangye | Shandan | No | 1 | 0.180505415 |
|  | Pingliang | Kongtong District | No | 3 | 0.541516245 |
|  | Pingliang | Chongxin | No | 1 | 0.180505415 |
|  | Pingliang | Zhuanglang | No | 3 | 0.541516245 |
|  | Qingyang | Qingcheng | No | 3 | 0.541516245 |
|  | Linxia Prefecture | kangle | No | 4 | 0.722021661 |
|  | Longnan | Kangxian | No | 3 | 0.541516245 |
|  | Tianshui | Zhangjiachuan | No | 1 | 0.180505415 |
|  | Longnan | Huixian | No | 1 | 0.180505415 |
|  | Longnan | Chengxian | No | 1 | 0.180505415 |
|  | Jiuquan | Dunhuang City | No | 7 | 1.263537906 |
|  | Baiyin | Jingtai | No | 1 | 0.180505415 |
|  | Lanzhou | Yuzhong | No | 3 | 0.541516245 |
|  | Tianshui | Wushan | No | 1 | 0.180505415 |
|  | Wuwei | Minqin | No | 1 | 0.180505415 |
|  | Jinchang | Yongchang | No | 1 | 0.180505415 |
|  | Lanzhou | Yongdeng | No | 3 | 0.541516245 |
|  | Linxia Prefecture | Yongjing | No | 4 | 0.722021661 |
|  | Pingliang | Jingchuan | No | 2 | 0.36101083 |
|  | Dingxi | Weiyuan | No | 4 | 0.722021661 |
|  | Dingxi | Zhangxian | No | 1 | 0.180505415 |
|  | Pingliang | Lingtai | No | 1 | 0.180505415 |
|  | Jiuquan | Yumen City | No | 2 | 0.36101083 |
|  | Gannan Prefecture | Maqu | No | 5 | 0.902527076 |
|  | Qingyang | Huanxian | No | 1 | 0.180505415 |
|  | Zhangye | Ganzhou District | No | 3 | 0.541516245 |
|  | Tianshui | Gangu | No | 2 | 0.36101083 |
|  | Baiyin | Silver District | No | 1 | 0.180505415 |
|  | Lanzhou | Gaolan | No | 1 | 0.180505415 |
|  | Tianshui | Qin'an | No | 2 | 0.36101083 |
|  | Zhangye | Sunan | No | 6 | 1.083032491 |
|  | Gannan Prefecture | Zhouqu | No | 1 | 0.180505415 |
|  | Longnan | Xihe | No | 2 | 0.36101083 |
|  | Gannan Prefecture | Diebu | No | 2 | 0.36101083 |
|  | Jiuquan | Quinta | No | 1 | 0.180505415 |
|  | Qingyang | Zhenyuan | No | 2 | 0.36101083 |
|  | Jiuquan | Aksai | No | 2 | 0.36101083 |
|  | Baiyin | Jingyuan | No | 1 | 0.180505415 |
|  | Pingliang | Jingning | No | 1 | 0.180505415 |
|  | Tianshui | Maiji District | No | 4 | 0.722021661 |
