# Supplementary figures and images for: Has the establishment of national parks improved nature-based tourism experiences? Evidence from social media data
Source: PLoS One. 2026 Mar 20;21(3):e0343256. doi: 10.1371/journal.pone.0343256 (PMC13004529; doi:10.1371/journal.pone.0343256)

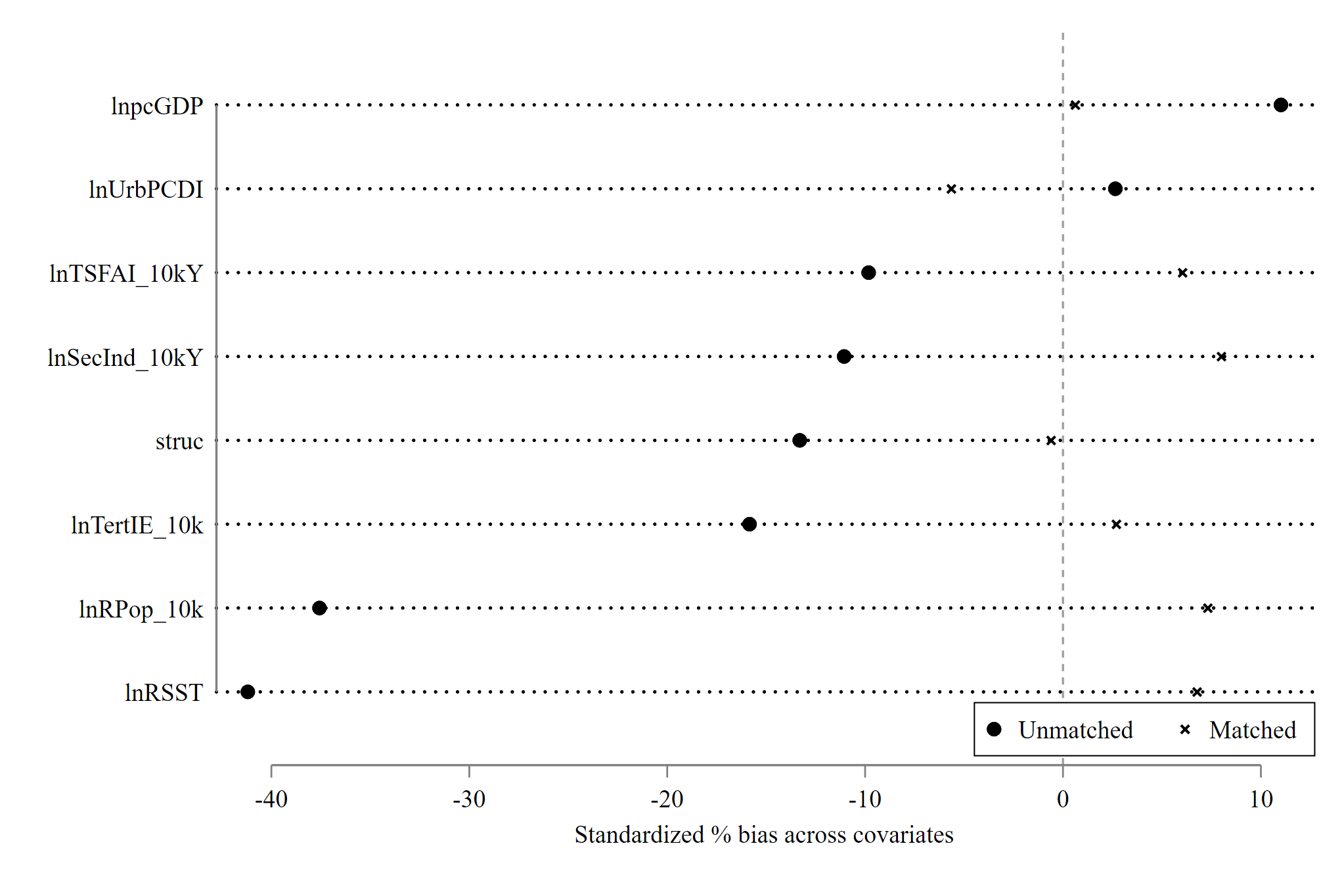

Supplement: S1 Fig — (TIF) [file pone.0343256.s007.tif]

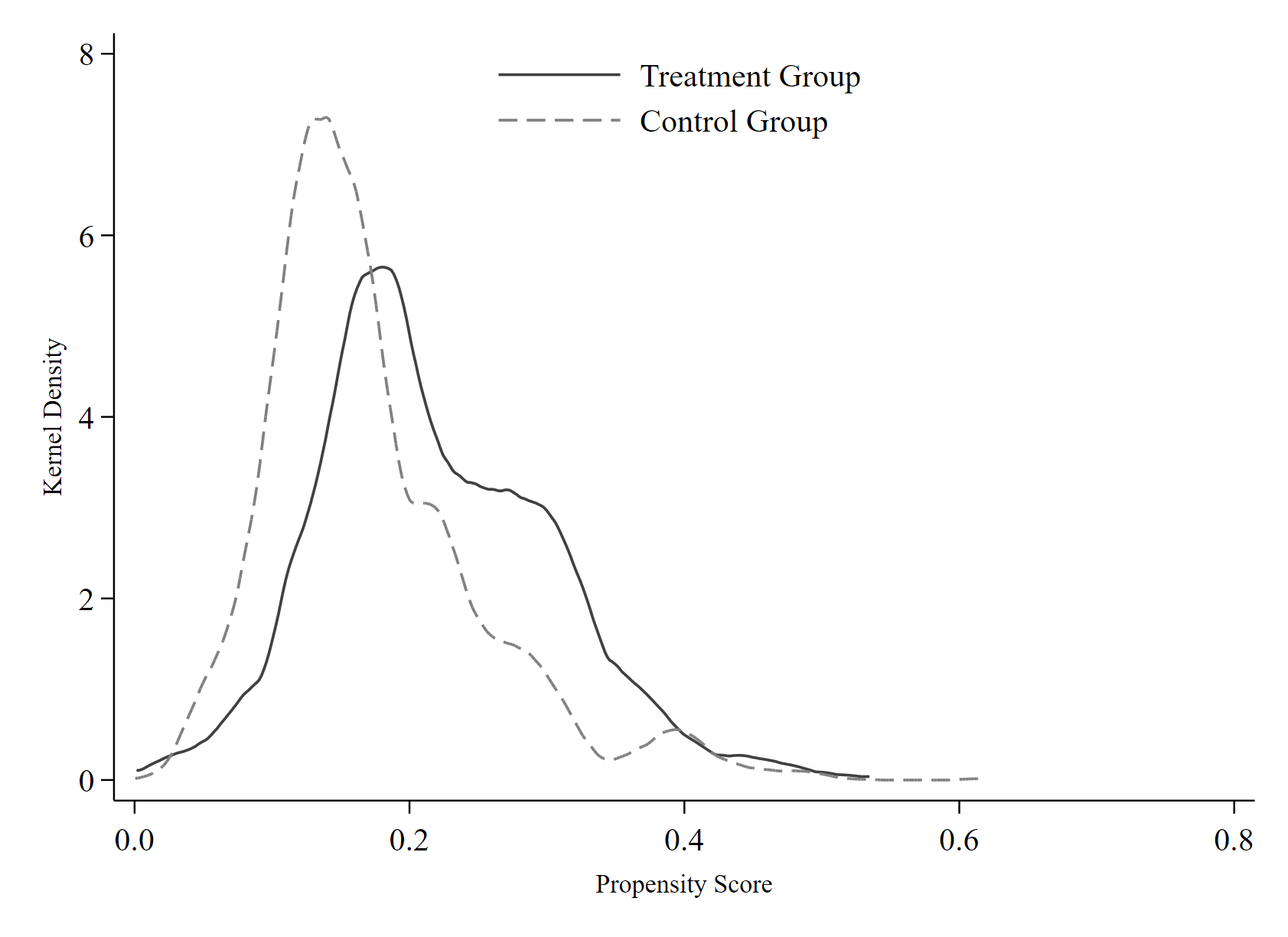

Supplement: S2 Fig — (TIF) [file pone.0343256.s008.tif]

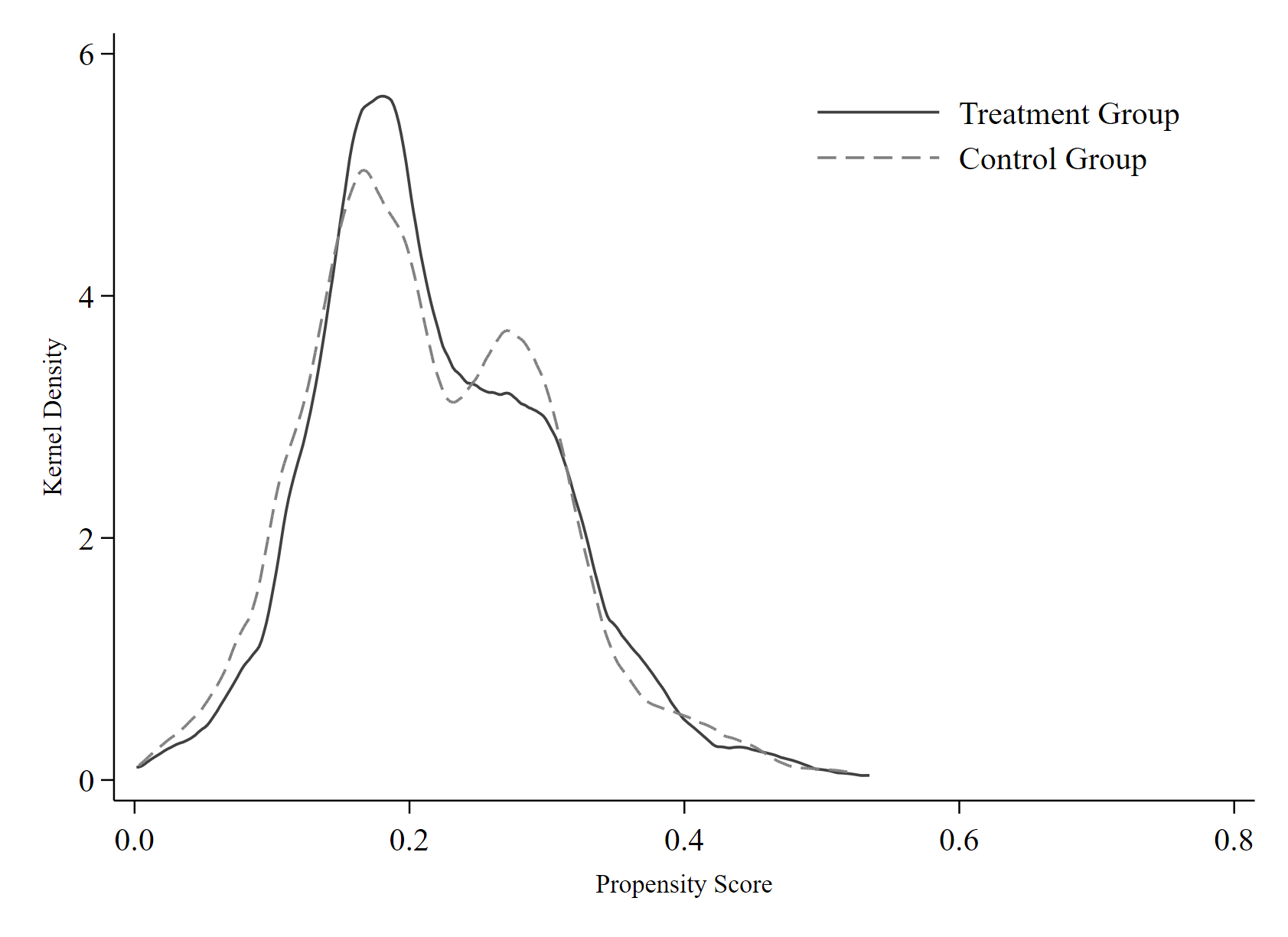

Supplement: S3 Fig — (TIF) [file pone.0343256.s009.tif]

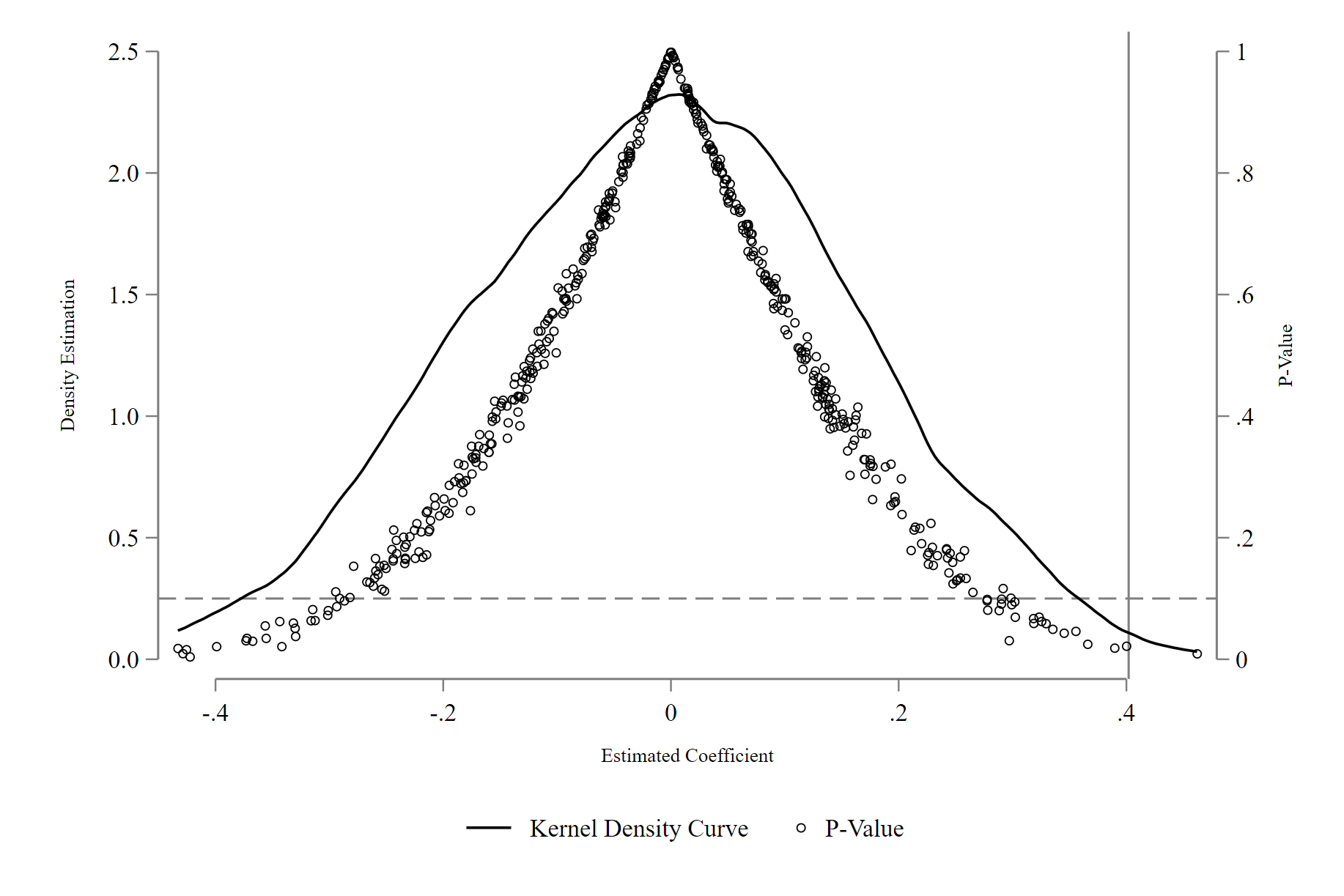

Supplement: S4 Fig — (TIF) [file pone.0343256.s010.tif]
